# Evaluation of the Effects of Two Different Feeding Frequencies on the Digestive Biochemistry of Two Mullet Species (*Chelon labrosus* and *Liza aurata*)

**DOI:** 10.3390/ani13020287

**Published:** 2023-01-13

**Authors:** Raquel Quirós-Pozo, Francisco Javier Moyano, Khalida Bainour, Sara Ramírez-Bolaños, Anais Ventura-Castellano, Javier Roo, Lidia Robaina

**Affiliations:** 1 Grupo de Investigación en Acuicultura, IU-ECOAQUA, Universidad de Las Palmas de Gran Canaria (ULPGC), 35214 Las Palmas, Spain; 2 Departamento de Biología y Geología, Facultad de Ciencias, Campus de Excelencia Internacional del Mar (CEI-MAR), Universidad de Almería, 04120 Almería, Spain

**Keywords:** Mugilidae, digestive physiology, feeding frequency, diversification, sustainable, aquaculture

## Abstract

**Simple Summary:**

Diversification of cultured fish species is one key measure to promote the sustainable development of marine aquaculture. Mullets (Mugilidae) present great potential due to their eurythermal, euryhaline, and low-trophic nature. However, there are many similar mullet species, and their physiological differences and optimal culture conditions are quite unknown, mainly when reared under intensive systems. In this sense, increasing the knowledge about changes in their digestive biochemistry related to feeding strategies is essential for a sustainable and cost-effective culture of these species. For these reasons, the present study aimed to characterize the digestive biochemistry of two mullet species (*Chelon labrosus* and *Liza aurata*) and to evaluate how it is affected when fish are fed using two different feeding frequencies: one or three times per day. In addition, changes in the potential bioavailability of nutrients determined by the two feeding patterns were assessed using digestive simulations performed in vitro. The results demonstrated the convenience of a feeding pattern of three meals per day instead of one. Moreover, the results evidenced that although *Chelon labrosus* and *Liza aurata* are species placed in a similar trophic level, their digestive response to feeding patterns, as well as body composition, present differences when fish are reared under the same conditions. This suggests that such interspecific variation must be considered when fish are reared under intensive conditions.

**Abstract:**

Mullets (Mugilidae) present significant potential for sustainable aquaculture diversification due to their eurythermal, euryhaline, and low-trophic nature. However, the physiological differences and optimal cultured conditions among the diverse mullet species are quite unknown. For these reasons, the present study aimed to address two main objectives: (1) to characterize the differences in digestive biochemistry, somatic indexes, and body composition between two mullet species (*Liza aurata* and *Chelon labrosus*); and (2) to evaluate the interactions of two different feeding frequencies (one against three meals per day) on the above-mentioned parameters, and also on the potential bioavailability of nutrients determined using in vitro assays. The results evidenced higher protease and amylase activities for *Chelon labrosus* than for *Liza aurata*, while the latter species presented a higher percentage of eviscerated weight and muscle lipids. Furthermore, the results from in vitro assays supported the higher enzyme activity of *Chelon labrosus* by an observed increase in the release of amino acids and reducing sugars measured for this species. Regarding feeding patterns, the results of the in vitro assays simulating enzyme: substrate (E:S) ratios corresponding to one or three meals per day point to a clear increase of nutrient bioavailability when the daily ration is split into several meals. The present results improve the physiological knowledge of mullet species and define criteria to develop better management protocols by producers.

## 1. Introduction

Global aquaculture production has more than tripled from 1997 to 2017 [1], reaching a historical record in 2020 with 122.6 million tons produced and a total value of USD 281,500 (United States Dollar), thus increasing the availability of aquatic foods to the population [2]. However, the diversity of products offered by aquaculture is still lower compared to those coming from fisheries due to different factors such as difficulties in the management of reproduction, production of suitable feeds, and other operational issues of the selected species. For this reason, diversification is one of the most critical challenges that aquaculture faces today. In the case of the EU (European Union), marine fish production is focused mainly on species belonging to high trophic levels, such as the seabass (*Dicentrarchus labrax*), or the turbot (*Scophthalmus maximus*). In contrast, the production of marine species placed at a low trophic level is still uncommon, despite their recognized importance for the sustainable development of aquaculture [3,4].

Nowadays, the EU’s efforts to increase aquaculture diversification include species such as the grey mullet (*Mugil cephalus*). Mullets are catadromous teleosts living in temperate waters worldwide, which can feed on a wide range of materials, including plankton, macroalgae, and detritus [5]. There are up to 71 recognized species [6] but only some of them are currently interesting for aquaculture, including the flathead grey mullet (*Mugil cephalus*), the thick-lipped grey mullet (*Chelon labrosus*), the golden grey mullet (*Liza aurata*), the thin-lipped grey mullet (*Liza ramada*), and the feral leaping mullet (*Liza saliens*), the most frequently farmed species in the Mediterranean region [7]. In 2020, 291.2 thousand tonnes of mullets were produced globally from aquaculture [2]; their traditional cultivation being carried out in ponds and estuaries due to their greatly appreciated flesh and processed products in many regions worldwide [8]. However, despite being cultured extensively or semi-intensively in many territories, there is still not enough information about their digestive capacity, nutritional requirements, or body composition when reared under intensive culture conditions.

Additionally, the natural co-existence of different mullet species in the same habitats has determined different feeding behaviours that help to avoid competition for similar resources [9]. This suggests that despite the important similarities between species, differences in key features related to food selection and other aspects of their digestive biochemistry and physiology must be considered when adopting suitable species-specific rearing practices. Among these, feed intake and feeding frequency are closely linked to the digestion transit rate, and, therefore, to the effectiveness of digestion.

Considering the above-detailed points, the aim of the present study was to (1) characterize differences in the digestive biochemistry (activities of alkaline protease and amylase), body composition, and somatic indexes between two similar mullet species (*Chelon labrosus* and *Liza aurata*) when reared under the same intensive culture conditions and (2) to evaluate the effects and interactions of two feeding frequencies (one and three meals per day) on the parameters mentioned above for both species. Both aspects were additionally connected using an in vitro assay simulating the digestion of proteins and carbohydrates under the conditions used in the in vivo experiment. Comparative information obtained may help optimize feed formulation and nutritional management for a more sustainable intensive culture of these species.

## 2. Materials and Methods

### 2.1. Fish and Facilities

The fish used in the present experiment were captured close to the Sports Pier of Las Palmas de Gran Canaria and then transported and acclimated in the GIA-ECOAQUA facilities, where they were maintained in 500-litre tanks in open-flow system conditions. The two species were identified and classified according to morphological parameters [10]. During this period, the fish were fed until apparent satiation twice per day with a commercial marine feed (Skretting). For each species, thirty-six juveniles (39.25 ± 5.28 g and 15.69 ± 0.86 cm of total weight and length, respectively) were distributed in six cylindrical tanks of 250 L (six fish per tank, twelve total tanks). The trial was run under an open seawater system and controlled photoperiod (12 h of light and 12 h of darkness), close to that naturally present in the season and latitude. Temperature and oxygen concentration were measured daily, and the average values were 20.95 ± 0.39 °C and 6.19 ± 0.28 mg/L, respectively.

### 2.2. Experimental Design

Fish were fed a 3 mm diet produced in the pilot plant of products and processes at GIA-ECOAQUA facilities in Taliarte, Gran Canaria (Spain), following the formula described for *Liza aurata* juveniles by Quirós-Pozo et al. [11] (Table 1), which adapted referenced levels of dietary protein and lipids for mullets [12,13,14]. The fish were fed at two different patterns that were established following the approximation described by Busti et al. [15], considering the maximum amount of food accepted in a single meal as a reference for the fixed daily ration of feed given to the animals. This dose, previously determined to be around 1% of the body weight, was then provided to triplicate groups of six fish in either one meal (8:00 h) or equally divided into three meals per day (8:00, 11:00, and 14:00 h). The feeding was performed carefully to avoid the loss of food. The experiment lasted 6 weeks to allow the fish to adapt to the new systems and feed and, thus, to better determine the digestive biochemistry adaptation to the feeding patterns. The fish were sampled at a middle point to adjust the fixed ration of offered feed. As the daily ration of feed was the same, feeding frequencies and species were chosen as the two variables under study.

After 6 weeks of feeding, fish samples were collected to measure the length of the digestion process and the total production of alkaline protease and amylase, which was the information required to develop the in vitro digestive simulation assay. For this, after a fasting period of 48 h, fish were fed following the two established patterns (one or three meals/day) and sampled at two different moments (15 and 40 h post-feeding) to check potential differences in enzyme activities between a fully established and almost finished digestion process. The manipulation and sacrifice of fish were rigorously conducted according to the European Union Directive (2010/63/EU) on animal welfare protection for scientific purposes. The experimental protocol performed for this work was approved by the Bioethical Committee of the University of Las Palmas de Gran Canaria (OEBA_ULPGC_09/2020).

Once sacrificed using immersion in ice-cold water, fish were measured and then dissected to separate the intestines. At that moment, body indexes were determined and samples from the liver and muscle were also conserved for biochemical analysis. The hepatosomatic index (HSI), percentage of eviscerated weight, and percentage of digestive tract weight were calculated with the following formulas:

Hepatosomatic index (HSI) (%) = liver weight (g)/fish weight (g) × 100.

Eviscerated weight (%) = eviscerated weight (g)/fish weight (g) × 100.

Digestive weight (%) = digestive tract weight (including perivisceral fat) (g)/fish weight (g) × 100.

### 2.3. Biochemical Assays

The muscle and liver samples (3 fish per tank) were pooled (9 fish and 3 pools per treatment) and stored at −80 °C until analysed. The analyses of the proximate composition of tissues and the experimental diet were performed following the techniques described in AOAC [16], with proteins being determined using the Kjeldahl technique [17], and the total lipids being determined following the protocol described by Folch et al. [18].

Crude enzyme extracts used for measuring enzyme activities and to develop the in vitro digestion assays were prepared with manual homogenization of the whole intestine and its contents in distilled water (1:10, weight/volume), followed by centrifugation (12.000 rpm, 3 °C, 15 min) (6 fish per tank and 3 pools in total per treatment). Total alkaline protease activity in the extracts was measured at pH 8.5 according to Kunitz’s method [19] which was modified by Walter [20] using casein as substrate. One unit of enzyme activity (U) was defined as the amount of enzyme needed to catalyse 1 μg of tyrosine formation per minute. Total amylase activity was measured at pH 7.5, following the 3,5-di-nitrosalicylic acid (DNSA) method [21], using starch as substrate. One unit of amylase activity was defined as the amount of enzyme needed to catalyse the formation of 1 μg of maltose equivalent per minute.

### 2.4. In Vitro Digestion Assays

The in vitro digestion assays were performed using membrane bioreactors modified from those described by Morales and Moyano [22]. The device consists of two chambers separated by a semipermeable membrane of 3500 kDa MWCO (Kilodalton, molecular weight cut-off). Fish enzyme extracts and feed samples were placed in the upper chamber and maintained under continuous agitation using a magnetic stirrer. Hydrolysis products (either amino acids or reducing sugars) passing across the membrane into the lower chamber were recovered at different time intervals during the reaction time. The whole system was maintained at 25 °C within a temperature-controlled chamber. The release of amino acids was quantified using the orthophthaldehyde (OPA) method described by Church et al. [23], and reducing sugars were measured using the DNSA method mentioned above. Some of the operating conditions used in the assays were maintained constant: 4 h total time, with samplings each hour, and pH = 8.2, while the enzyme: substrate ratio (E:S) was adapted to simulate the expected changes correlated to the different feeding patterns. This physiological E:S ratio (Table 2) was estimated only for protease. It was determined considering, on the one hand, the total enzyme production calculated for the average size of the individuals used in the experiment, and, on the other hand, the average intake of protein ingested when the fish received the amount of feed corresponding to the daily ration either in one or three meals. Hence, in the assays, a fixed amount of enzyme extracts was combined with two different amounts of the substrate assuming that, in the live fish, enzyme secretion as a response to intake is independent of the quantity of feed ingested.

### 2.5. Statistical Analysis

After completing normality and homoscedasticity tests, data obtained in the different experiments were evaluated using a two-way ANOVA (species (S) and feeding frequency (F)) followed by a “least significant difference” (LSD) test or a Kruskal–Wallis’s test in cases where the data did not meet normality or homoscedasticity. All analyses were carried out using the program IBM^®^ SPSS Statistic 20 (New York, NY, USA). In addition, the statistical power analysis program G*power [24] was used to determine the minimum number of fish required to run the experiment.

## 3. Results

### 3.1. Somatic Indexes

The results of somatic indexes are shown in Table 3. Significant differences were evidenced between both species, with a higher liver and digestive tract size measured in *C. labrosus* and a higher percentage of eviscerated weight for *L. aurata*. Additionally, mullets fed once daily presented a higher weight percentage of the digestive tract.

### 3.2. Biochemical Assays

The proximate composition of the muscle and liver is detailed in Table 4. The muscle lipid content was significantly higher in *L. aurata* than in *C. labrosus*. The liver lipid content was significantly higher in fish receiving their daily ration in only one meal.

### 3.3. In Vitro Digestion Assays

**Figure 1:**
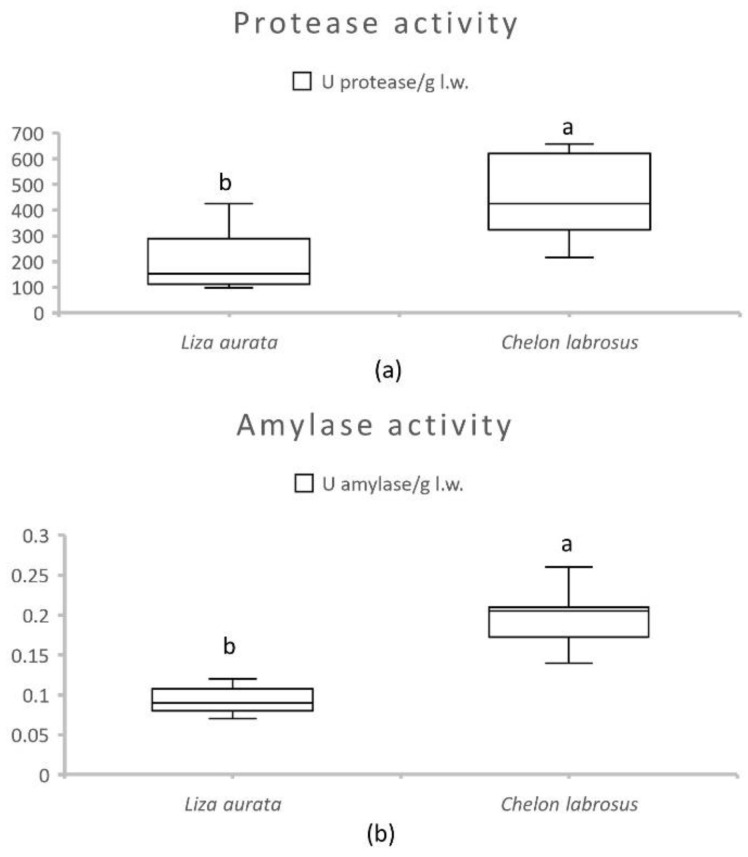
Average activity of total alkaline protease (**a**) and amylase (**b**) (U/g live weight) in the intestines of *C. labrosus* and *L. aurata* juveniles (n = 36). Data expressed as means ± standard deviation (SD). Significant differences between species (*p* < 0.05) are indicated using lowercase letters (a, b).

Activities of intestinal alkaline protease and amylase are presented in Figure 1 for both species. The results showed significantly higher values for both enzymes in samples from *C. labrosus* (*p* ≤ 0.01). The effect of the number of daily meals and sampling moments on the enzyme activities of each species is shown in Table 5. In *L. aurata*, the protease activity was significantly decreased (*p* = 0.01) with time spent after the first meal. In contrast, the intestinal protease activity in *C. labrosus* did not show significant variations related to the feeding frequency or sampling moment. This was also the case for amylase activity in both species.

**Figure 2:**
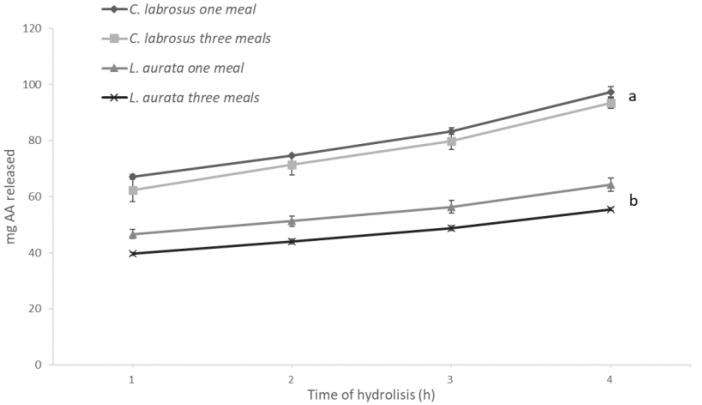
Release of amino acids (AA) from *L. aurata* and *C. labrosus* when simulating the different feeding frequencies in vitro (n = 18). Data expressed as means ± SD. Significant differences between species (*p* < 0.05) are indicated using lowercase letters (a, b).

**Figure 3:**
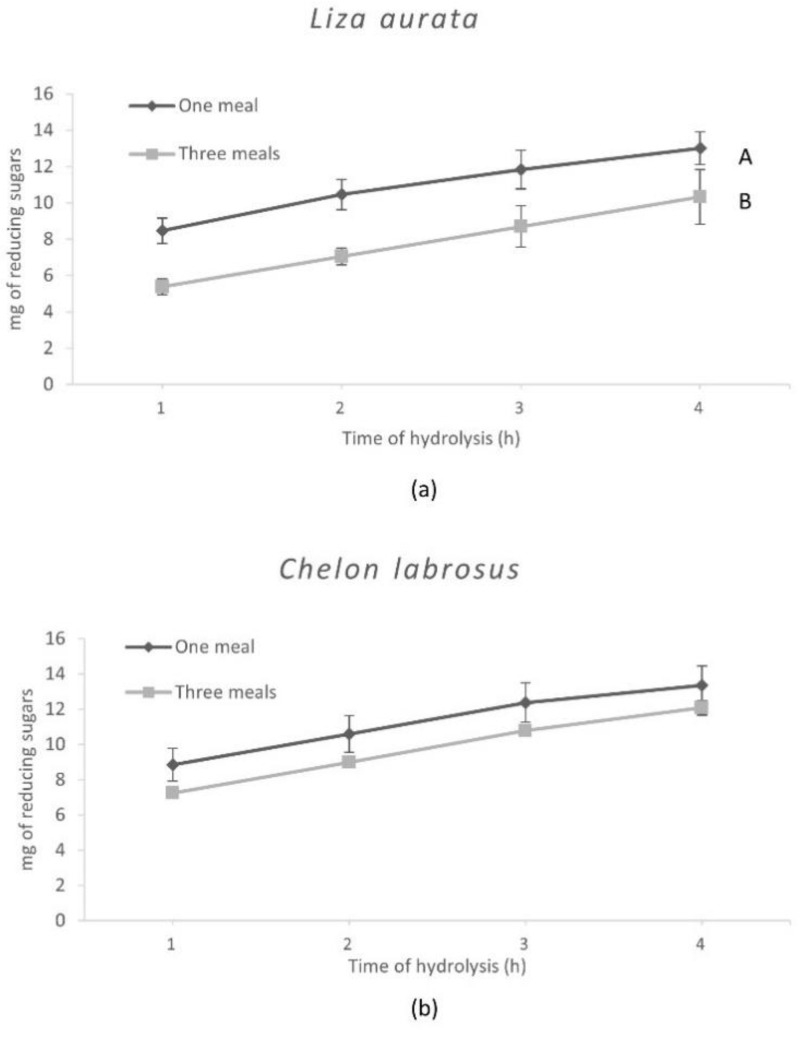
Release of reducing sugars from *L. aurata* (**a**) and *C. labrosus* (**b**) when the different feeding frequencies in vitro (n = 18). Data expressed as means ± SD. Significant differences between feeding patterns (*p* < 0.05) are indicated using uppercase letters (A, B).

The results obtained with the in vitro assays are shown in Figure 2 and Figure 3 and reflect the specific differences observed in the enzyme activity levels for both species. Hence, after hydrolysis, the release of amino acids was significantly higher (*p* = 0.04) when simulating digestion in *C. labrosus* than in *L. aurata*. On the other hand, the total release of amino acids was not significantly affected by the different E:S ratios used to simulate the conditions associated with the two feeding patterns in both species. In contrast, a significantly lower release of reducing sugars was obtained when testing an E:S ratio adapted to simulate three meals in *L. aurata*, but this was not the case in *C. labrosus*.

## 4. Discussion

The low voluntary intake in a single meal observed in both species can be partially explained by considering that mullets are continuous feeders. Their stomachs are formed by a thin-walled cardiac and a thick-walled pyloric portion [25], which is adapted to function as a mill for feed particles, similar to the gizzards of birds. For this reason, such a stomach is much less distensible than that present in other fish species with a more discontinuous feeding pattern, which limits to a great extent the maximum amount of feed that mullets can ingest in one meal [26]. This could explain why, in the present experiment, the maximum intake in one single meal used as a reference for the two selected feeding patterns accounted for only 1% of the body weight.

With regards to biochemical composition, interestingly, muscle from *L. aurata* juveniles presented a higher percentage of lipids than *C. labrosus*, which is a valuable feature due to the beneficial role of marine products from aquaculture in human nutrition and health [27,28], especially for its polyunsaturated fatty acid content. In the case of the livers, many fish species are known to store lipids in this organ, which is sensitive to feeding management [29]. In the present trial, fish fed only once per day presented a higher amount of liver lipids, indicating that these animals could accumulate energy reserves in the form of fat when food is offered infrequently. Furthermore, the higher weight percentage of the digestive tract (produced probably by a higher amount of perivisceral fat) in mullets fed once per day supports this hypothesis. On the other hand, the lower proportion of viscera in relation to the carcass of *L. aurata*, in addition to a more stylized body, gives an interesting benefit to increasing the culture of these animals.

Essential differences in the use of nutrients are determined by specific features of the digestive biochemistry of fish species [30,31]. In the present study, significantly higher total activities of both protease and amylase were measured in *C. labrosus* compared to *L. aurata*. This suggests a higher potential for the hydrolysis of essential nutrients in the former species that could reflect differences in the proximate composition of the food items that constitute its diet under natural conditions. In the present study, two factors may influence the measured total activities of digestive enzymes: the number of daily meals and the moment of sampling. In this sense, lower enzyme activities could be expected when fish are fed under suboptimal feeding frequencies [32]. On the other hand, since digestive enzymes are partially hydrolysed and eliminated with faeces as digestion progresses [33], samplings done at different moments after a meal should evidence a decreasing trend in the activities. In the present study, the number of daily meals and sampling moments influenced enzyme activities differently in both species. In *C. labrosus*, no significant variations in the total activities of protease or amylase were detected irrespective of the amount of food ingested or the sampling moment. This suggests the existence of a pattern of enzyme production frequently described in continuous feeders, in which the maintained presence of food in the gut is associated with a continuous secretion of the enzymes, ensuring suitable hydrolysis of the primary substrates [34]. This maintained production of intestinal proteases is also a typical feature described in stomachless fish as a compensatory mechanism for the absence of acid hydrolysis of proteins [35]. In the case of *L. aurata*, the observed reduction in the total activity of alkaline protease linked to the digestion time points suggests an enzyme production pattern less identifiable compared to that of a continuous feeder. However, the observed variations cannot be fully assigned to an occasional feeder.

Both species have been widely described as omnivorous and opportunistic fishes having high trophic overlap [36], analogous trophic niche breadths [37], and similar relative gut lengths [27]. However, for different seasons and estuarine systems, a slightly higher trophic level has been described for *L. aurata* (3.02–3.30) than for *C. labrosus* (2.16–3.29) [9]. In addition to the differences found in digestive biochemistry, this point contributes to differentiating physiological strategies in each species that can be driven by the differences in the composition of the food items they preferably consume in the wild.

Regarding the results obtained with the in vitro simulation of digestion, the higher protease activity measured in the digestive tract of *C. labrosus* determined a significantly higher release of amino acids from the same amount of substrate than that of *L. aurata*. Surprisingly, no significant differences were obtained in the total amount of amino acids released when simulating the two different enzyme: substrate ratios theoretically present in the guts of fish ingesting their daily ration either in one or three meals. In the first case, the expected net release of amino acids from the feed should have been higher than when three times less substrate was present. This points out that in the case of intestinal proteases, an optimal enzyme: substrate ratio is reached when a very low amount of substrate is present and suggests that, from a practical point of view, it should be better to distribute the daily ration into several meals. Results obtained for carbohydrate hydrolysis in *C. labrosus* confirm this suggestion and are in concordance with those obtained in juveniles of *Mugil liza*, in which a higher feed efficiency was obtained when fish were fed three times per day [38]. In addition, Solovyev and Gisbert [39] recorded inappropriate enzyme/substrate ratios and lower digestive efficiency and growth rates in *Mugil cephalus* fry fed one or two times per day, compared to mullet fry provided a continuous regime. Similar results have also been described in other species such as the snapper (*Lutjanus johnii*) [40] or the doctor fish (*Garra rufa*) [41].

## 5. Conclusions

To summarize, concerning the comparison between feeding frequencies, the results obtained in the digestion model support the observed benefits for three daily meals instead of one. Regarding the comparison between the two species, *L. aurata* showed higher muscle lipid content compared to *C. labrosus*, in addition to a greater eviscerated weight and lower HSI. *C. labrosus* instead presented higher protease and amylase activities which determined an increased release of amino acids and reducing sugars in the in vitro assays. The obtained results suggest the different potentialities of these similar fish species when reared under diverse circumstances and open the future to species-specific nutrition and feeding research among others.

## Figures and Tables

**Table 1:** Ingredients (g/100 g) and proximate composition of the diet used in the experiment.

Ingredients (%)	
Fish meal (Peruvian origin) ^a^	20.0
Blood meal ^b^	3.0
Ulva meal ^c^	10.0
Rapeseed meal (0.0) ^d^	8.0
Corn meal ^e^	6.0
Wheat gluten ^e^	6.0
SPC (soy protein concentrate) ^f^	20.0
Wheat meal ^e^	6.0
Fish oil ^a^	8.5
Soy lecithin ^g^	1.0
Vitamin mix ^h^	2.0
Mineral mix ^i^	2.0
Ca(H_2_PO_4_) _2_^j^	1.0
CMC ^k^ **Analytical composition (% dry weight)**	0.5
Protein	40.9 ± 0.4
Lipids	14.1 ± 0.6
Ash	11.4 ± 0.1
Moisture	8.1 ± 0.6

^a^ Fish meal and fish oil from South America (supplied by Skretting, Spain); ^b^ Blood meal (supplied by Dibaq, Spain); ^c^ Ulva meal (supplied by Puerto Muiños S.L., Spain); ^d^ Rapeseed 0.0 (supplied by Dibaq, Spain); ^e^ Flours obtained from local producers; ^f^ Soy protein concentrate (Sopropeche, France); ^g^ Soy lecithin (92% fat) (supplied by Korott S.L., Spain); ^h^ Vitamin premix containing (mg kg-^1^ or IU/kg of dry feed): thiamine 40 mg, riboflavin 50 mg, pyridoxine 40 mg, calcium pantothenate 117 mg, nicotinic acid 200 mg, folic acid 10 mg, cyanocobalamin, 0.5 mg, choline chloride 2700 mg, myo-inositol 2000 mg, ascorbic acid 5000 mg, menadione 20 mg, cholecalciferol 2000 IU, ethoxyquin 100 mg, retinol acetate 5000 IU, vitamin E (DL-alpha-tocopherol acetate) 250 mg; ^i^ Mineral premix containing (g/kg of dry feed): calcium orthophosphate 1.60 g, calcium carbonate 4 g, ferrous sulphate 1.5 g, magnesium sulphate 1.6 g, potassium phosphate 2.8 g, sodium phosphate 1 g, aluminium sulphate 0.02 g, zinc sulphate 0.24 g, copper sulphate 0.20 g, manganese sulphate 0.08 g, potassium iodate 0.02 g; ^j^ Sigma-Aldrich, Munich, Germany; ^k^ carboxymethylcellulose (sodium salt, Sigma-Aldrich, Munich, Germany).

**Table 2:** Enzyme: substrate ratios used in the in vitro assays.

Species	Total U × 10^3^/Fish	Meals/Day	Food Intake Per Meal (mg)	Protease E:S Ratio (U/mg Food)	Amylase E:S Ratio (U/mg Food)
***L. aurata***	7.22 ± 3.65	1	350	21.82 ± 13.85	0.01 ± 0.00
		3	120	50.18 ± 21.15	0.03 ± 0.00
***C. labrosus***	15.72 ± 5.51	1	350	40.79 ± 2.27	0.02 ± 0.00
		3	120	142.08 ± 54.26	0.06 ± 0.00

Data expressed as means ± standard deviation (SD).

**Table 3:** Somatic indexes expressed by species (S) and feeding frequency (F) (n = 18).

	*L. aurata*		*C. labrosus*		*p* Values		
	One Meal	Three Meals	One Meal	Three Meals	S	F	S × F
%Eviscerated weight	87.38 ± 0.17	90.24 ± 1.58	86.55 ± 2.55	86.51 ± 0.64	0.02	0.10	0.10
HSI	0.83 ± 0.11	0.82 ± 0.03	1.04 ± 0.07	0.97 ± 0.04	0.00	0.30	0.41
% Digestive weight	7.02 ± 0.64	5.99 ± 0.37	11.16 ± 0.38	9.96 ± 1.11	0.00	0.04	0.82

Data expressed as means ± standard deviation (SD).

**Table 4:** Proximal composition (% of dry weight) of the muscle and liver expressed by species (S) and feeding frequency (F) (n = 9).

	*L. aurata*		*C. labrosus*		*p* Values		
	One Meal	Three Meals	One Meal	Three Meals	S	F	S × F
*Muscle*							
Lipid	16.49 ± 0.69	18.97 ± 3.19	13.18 ± 1.58	13.15 ± 1.06	0.00	0.29	0.21
Protein	80.83 ± 1.89	83.26 ± 4.82	84.46 ± 0.40	87.52 ± 2.40	0.08	0.08	-
Ash	6.89 ± 0.05	6.13 ± 0.53	6.00 ± 0.72	6.18 ± 0.62	0.08	0.26	-
*Liver*							
Lipid	40.22 ± 4.56	31.53 ± 5.53	44.14 ± 3.37	38.94 ± 5.33	0.61	0.03	0.52

Data expressed as means ± standard deviation (SD).

**Table 5:** Protease and amylase activities (U/g live weight) of *C. labrosus* and *L. aurata* as a function of the number of daily meals and sampling moment, expressed as sampling moment (SM) and feeding frequency (F) (n = 18).

	Feeding Frequency (F)		Sampling Moment (SM)		*p* Values		
	One Meal	Three Meals	15 h	40 h	F	SM	F × SM
***C. labrosus***							
Protease	411.35 ± 129.03	487.13 ± 185.88	500.68 ± 176.49	397.80 ± 130.95	0.25	0.13	0.01
Amylase	0.18 ± 0.03	0.21 ± 0.03	0.20 ± 0.03	0.20 ± 0.04	0.16	0.99	0.65
***L. aurata***							
Protease	218.22 ± 133.47	172.05 ± 72.52	270.25 ± 101.8	120.02 ± 21.01	0.26	0.01	0.13
Amylase	0.09 ± 0.03	0.10 ± 0.01	0.11 ± 0.03	0.09 ± 0.02	0.69	0.15	0.12

Data expressed as means ± standard deviation (SD). Data on enzyme activities as a function of feeding frequency is calculated irrespectively of the sampling moment and vice versa.

## Data Availability

Not applicable.
